# [Retracted] miR-372 promotes breast cancer cell proliferation by directly targeting LATS2

**DOI:** 10.3892/etm.2025.12926

**Published:** 2025-07-21

**Authors:** Xueyuan Cheng, Junqiang Chen, Zhong Huang

Exp Ther Med 15:2812–2817, 2018; DOI: 10.3892/etm.2018.5761

Following the publication of this paper, it was drawn to the Editor's attention by a concerned reader that the flow cytometric data shown in Fig. 3C on p. 2815 were strikingly similar to data that had already appeared in different form in another article written by different authors at different research institutes, which had already been published in the journal *OncoTarget*. After having performed an independent analysis of the data in the Editorial Office, it came to light that certain of the colony-formation assay data (the ‘Control’ data) in Fig. 2C on p. 2814 were also included in an article submitted for publication to another journal at around the same time.

In view of the fact that the abovementioned data had already apparently been published previously, the Editor of *Experimental and Therapeutic Medicine* has decided that this paper should be retracted from the Journal. After having been in contact with the authors, they accepted the decision to retract the paper. The Editor apologizes to the readership for any inconvenience caused.

